# The construction and analysis of tricarboxylic acid cycle related prognostic model for cervical cancer

**DOI:** 10.3389/fgene.2023.1092276

**Published:** 2023-03-09

**Authors:** Guanqiao Chen, Xiaoshan Hong, Wanshan He, Lingling Ou, Bin Chen, Weitao Zhong, Yu Lin, Xiping Luo

**Affiliations:** ^1^ Guangzhou Medical University, Guangzhou, China; ^2^ Department of Gynecology, Guangdong Women and Children Medical Hospital, Guangzhou, China; ^3^ Department of Surgical Neonatal Intensive Care Unit, Guangzhou Women and Children’s Medical Center, Guangzhou, China; ^4^ Nanfang Hospital, Southern Medical University, Guangzhou, China; ^5^ Guangzhou University of Chinese Medicine, Guangzhou, China

**Keywords:** cervical cancer, tricarboxylic acid cycle (TCA cycle), metabolic reprogramming, prognostic signature, bioinformatics

## Abstract

**Introduction:** Cervical cancer (CC) is the fourth most common malignant tumor in term of in incidence and mortality among women worldwide. The tricarboxylic acid (TCA) cycle is an important hub of energy metabolism, networking one-carbon metabolism, fatty acyl metabolism and glycolysis. It can be seen that the reprogramming of cell metabolism including TCA cycle plays an indispensable role in tumorigenesis and development. We aimed to identify genes related to the TCA cycle as prognostic markers in CC.

**Methods:** Firstly, we performed the differential expressed analysis the gene expression profiles associated with TCA cycle obtained from The Cancer Genome Atlas (TCGA) database. Differential gene list was generated and cluster analysis was performed using genes with detected fold changes >1.5. Based on the subclusters of CC, we analysed the relationship between different clusters and clinical information. Next, Cox univariate and multivariate regression analysis were used to screen genes with prognostic characteristics, and risk scores were calculated according to the genes with prognostic characteristics. Additionally, we analyzed the correlation between the predictive signature and the treatment response of CC patients. Finally, we detected the expression of ench prognostic gene in clinical CC samples by quantitative polymerase chain reaction (RT-qPCR).

**Results:** We constructed a prognostic model consist of seven TCA cycle associated gene (ACSL1, ALDOA, FOXK2, GPI, MDH1B, MDH2, and MTHFD1). Patients with CC were separated into two groups according to median risk score, and high-risk group had a worse prognosis compared to the low-risk group. High risk group had lower level of sensitivity to the conventional chemotherapy drugs including cisplatin, paclitaxel, sunitinib and docetaxel. The expression of ench prognostic signature in clinical CC samples was verified by qRT-PCR.

**Conclusion:** There are several differentially expressed genes (DEGs) related to TCA cycle in CC. The risk score model based on these genes can effectively predict the prognosis of patients and provide tumor markers for predicting the prognosis of CC.

## Introduction

Cervical cancer (CC) is the fourth most common cancer and also the fourth leading cause of cancer related deaths. According to a report released by the International Agency for Research on Cancer (IARC) in 2018, there are 570,000 new cases and 310,000 deaths in the world in this year (CA Cancer J Clin, 2020). In spite of the promotion of the HPV-related vaccine and screening programs, many patients with CC are already advanced or have locally advanced cancer at diagnosis, which leads to a poor prognosis. Previous studies found that 5-year survival rate of CC patients detected at an early stage is 92% (Bray et al., 2018), whereas the 5-year survival rate for advanced CC patients, especially for metastatic CC patients, whose survival rates range from 5% to 15%, is still low (Moore, 2006). In order to improve survival rates, primary screening and early detection of CC are high priorities. The appropriate biomarkers for clinical diagnosis and prognosis have not been identified yet. Thus, better prognostic biomarkers for CC development are urgently required to increase patient survival.

As a central pathway of cellular oxidative phosphorylation, the TCA cycle participates in physiological processes such as cellular bioenergetics, biosynthesis, and REDOX balance. Most cancers, including CC, are a disease characterized by the accumulation of genetic alterations and genetic dysregulation, leading to uncontrolled cell proliferation requiring increased energy production and macromolecular synthesis (DeBerardinis and Chandel, 2016). In response to increased metabolic stress, malignant cells often reprogram their biochemical pathways so that nutrients can be rapidly absorbed and broken down, thereby promoting disease transformation, maintenance, and progression. As it is universally accepted that cancer cells primarily use aerobic glycolysis for respiration, the TCA cycle has been overlooked until recently in cancer metabolism and tumorigenesis. With modern technological advances such as unbiased and targeted metabolomics along with high-throughput sequencing technology, there are a wealth of new discoveries in the field of tumor metabolism. Recent studies have found that gankyrin positively regulates TIGAR transcription to promotes hepatocellular carcinoma progression by accelerating the conversion of glucose metabolism to PPP and TCA cycle (Yang et al., 2022). Furthermore, glutamine has been shown to be an indispensable nutrient source in many cancer types, particularly MYC-driven cancers (DeBerardinis and Cheng, 2010). Researchers pay increasing attention to lipid metabolism in tumorigenesis recent years. To sum up, these studies have provided compelling evidence that the TCA cycle serves as a significant role in cancer metabolism and tumorigenesis (Sajnani et al., 2017).

In this study, we conducted a series of analysis including Cox regression, LASSO regression and multivariate Cox regression based on TCA cycle-associated genes in CC. A prognostic risk model based on 7 gene signatures was constructed *via* TCGA database and externally validated by Gene Expression Omnibus (GEO) database. In the meanwhile, the model provided an indication of prognosis, diagnostic value and predicting response to chemotherapy for CC.

## Materials and methods

### Data collection and preprocessing

From TCGA database (https://portal.gdc.cancer.gov/), we obtained RNA sequence transcriptome data and relevant clinical information of 304 patients with CC and 3 normal adjacent tissue samples. From the GEO database (https://www.ncbi.nlm.nih.gov/geo/, GSE44001), we downloaded RNA sequencing data and clinical information of 300 patients for external validation.

### Identification of differentially expressed TCA cycle-related genes

The list of TCA cycle-related genes was obtained through literature mining (Arnold et al., 2022). Their mRNA expression levels between CC and normal adjacent tissue samples were compared according to TCGA cohort. The limma software package was used to identify the differentially expressed TCA cycle-related genes with the significance threshold (*p* < 0.05 and |log_2_FC|>1.5), which were presented as a heatmap. The “corrplot” package was used to reveal correlations between DEGs associated with the TCA cycle. An interaction network of proteins among TCA cycle-related DEGs was constructed using STRING and visualized using Cytoscape 3.8.0.

### Consensus clustering

The “ConsensuClusterPlus” R package was used for the analysis the comprehensive expression of the 18 differentially expressed TCA cycle-related genes to identify distinct subgroups of 302 CC samples. It was repeated 1,000 times to ensure classification stability (parameters: clustering algorithm, k-means; distance, Euclidean). The optimal k value was determined based on cumulative distribution function and delta area values. Principal Component Analysis (PCA) were performed by “Rtsne” R package to reduce the dimension of the 18 DEGs. The Kaplan-Meier method and log-rank test were used to evaluate the overall survival (OS) rate of patients with different subtypes. Chi-square test was used to analyze the distribution of age, tumor grade, tumor stage and histological type among different clusters.

### Construction of TCA cycle related prognostic signature

To sort out TCA cycle related genes with potential prognostic value (*p* < 0.05), univariate Cox analysis was performed for OS. Next, using a least absolute shrinkage and selection operator (LASSO) regression model, the optimal value was determined to build a prognostic gene signature. We used R’s glment package to perform Cox regression analysis and LASSO. On the basis of the following formula: Risk score = ∑Coefgene × Expgenes, risk scores for every single patient was calculated, where Coefgene represents the coefficient of each prognostic gene and Expgenes represents the expression level of each gene. According to the median risk scores, patients were divided into high-risk and low-risk group. In addiction, we plotted the receiver operating characteristic (ROC) curves and Kaplan–Meier plots. To perform the validation of the prognostic model, GEO dataset (GSE44001) was analyzed the prognostic value with similar methods.

### Functional Enrichment Analysis and cuproptosis-related gene analysis

Gene set enrichment analysis (GSEA) (https://www.broadlnstitute.org/gsea/) was used to identify differential expressions of genes (gene sets) that were functionally related and whose enrichment in CC patient subgroups was significant. Kyoto Encyclopedia of Genes and Genomes (KEGG) pathway databases were downloaded from the molecular signatures database (MSigDB) as the functional enrichment reference set (http://www.gsea-msigdb.org). Finally, the significantly enriched KEGG pathways are shown centrally. Gene set variation analysis (GSVA) was implemented in the “GSVA” R package to investigate potential molecular characteristics that differed between high- and low-risk groups. Access to the MSigDB, the Hallmark gene set “c2. cp.kegg.v2022.1. Hs.symbols.gmt” was gotten to applied in GSVA. According to a threshold of |log_2_FC| > 0.1 and *p* < 0.05, DEGs between high- and low-risk groups were screened and undergone to gene ontology (GO) and KEGG analyses using the “clusterProfiler” R package. Furthermore, we made a comparation the expression levels of cuproptosis-related genes between the high-risk group and the low-risk group, and visualized them by box plots.

### Immune checkpoint analysis and the role of the predictive signature in predicting the clinical treatment response

Spearman correlation analysis was performed using “cor.test” in R to analyze the correlation between immune checkpoint expression with *p* < 0.05 as the cutoff for significance. The pRRophetic R package was used to predict chemosensitivity based on data from the Genomics of Drug Sensitivity in Cancer pharmacogenomics database. The half maximal inhibitory concentration (IC50) of clinically commonly used chemotherapy drugs was calculated to evaluate the role of predictive signatures in predicting the treatment response of CC. We compared the IC50 values between the high- and low-risk groups *via* Wilcoxon signed-rank test.

### Analysis of quantitative reverse TranscriptionPolymerase chain reaction (qRT-PCR)

Both cervical cancer and adjacent non-cancerous tissues used in this study were obtained from postoperative patients with cervical cancer from 2019 to 2022 in Department of gynecology, Guangdong Women and Children Medical Hospital. All samples were obtained through review by the ethics committee, and the informed consent of CC patient was acquired. We extracted RNA from specimens by utilizing the TRIzol reagent (Ambion, United States), followed by reverse transcription into cDNA utilizing the QuantiTect Reverse Transcription Kit (Promega, United States). Quantitative PCR (qPCR) is a technique for measuring the amount of DNA present in a sampl in real time. With the aid of SYBR-Green (Vazyme, China), real-time qPCR assays were carried out, and expression levels were standardized to beta-actin levels. The sequences of primers are listed in Table 1.

**TABLE 1:** Consists of a collection of primer sequences utilized in this study.

Primer	Sequence (5′to 3′)
ACSL1-F	CTT​ATG​GGC​TTC​GGA​GCT​TTT
ACSL1-R	CAA​GTA​GTG​CGG​ATC​TTC​GTG
ALDOA-F	CAG​GGA​CAA​ATG​GCG​AGA​CTA
ALDOA-R	GGG​GTG​TGT​TCC​CCA​ATC​TT
FOXK2-F	GGA​GGC​GTC​TGA​GTC​TCC​A
FOXK2-F	CCC​ACC​TTG​TAC​CCT​GAA​GA
GPI-F	CCG​CGT​CTG​GTA​TGT​CTC​C
GPI-R	CCT​GGG​TAG​TAA​AGG​TCT​TGG​A
MDH1B-F	CTA​GCA​TGA​CGA​CTG​AAC​TGA​TG
MDH1B-R	AGA​GGC​ACT​GGT​GAT​CCA​GA
MDH2-F	TCG​GCC​CAG​AAC​AAT​GCT​AAA
MDH2-R	GCG​GCT​TTG​GTC​TCG​ATG​T
MTHFD1-F	GCG​CCA​GCA​GAA​ATC​CTG​A
MTHFD1-R	AGG​TAC​TTG​CTC​CTT​CAA​CTG​A
Beta-actin-F	GTG​AAG​GTG​ACA​GCA​GTC​GGT
Beta-actin-R	AAG​TGG​GGT​GGC​TTT​TAG​GAT

### Statistical analysis

All statistical analyses were performed with the use of R software (Version 4.2.1). Wilcoxon test was used to analyze the difference in the expression of TCA-related genes between normal and tumor tissues. Cox regression model was used for univariate and multivariate survival analysis to screen independent prognostic signature. The OS of patients in the high and low risk groups was analyzed by the Kaplan-Meier method and log-rank test. The ROC curve was drawn and the area under the curve (AUC) was determined by using the “survivalROC” software package. GraphPad Prism 9 program was used to draw scatter plots, and paired *t*-test was used to detect the differences in the expression of prognostic related genes between cervical cancer tissues and adjacent tissues. A *p*-value of less than 0.05 was considered to be statistically significant (*p* < 0.001 = ***, *p* < 0.01 = **, and *p* < 0.05 = *).

## Results

### Identification of TCA cycle-related DEGs between normal and CC tissues

**FIGURE 1:**
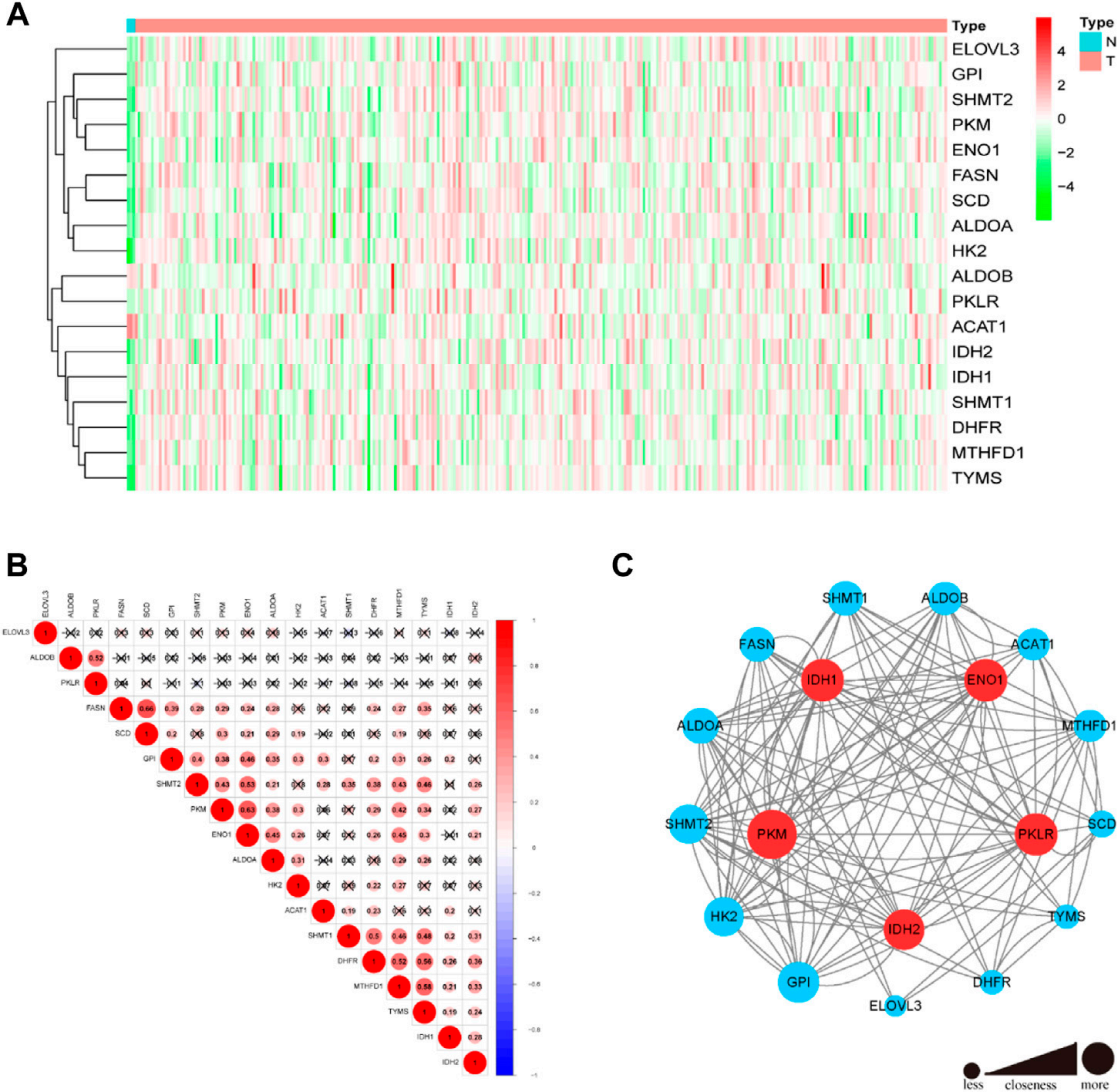
Differential expressions of 18 TCA cycle-related genes and tumor subclusters based on the TCA cycle-related DEGs. **(A)** The heatmap showed the 18 TCA cycle-related genes in tumor and normal adjacent tissues. **(B)** Display of the relationship between the TCA cycle-related DEGs. **(C)** PPI network indicated the interactions of the TCA cycle-related genes. (Red and green colors represent >0.65 and < = 0.65 closeness respectively).

A list of 117 TCA cycle-related genes was identified (Supplementary Table S1), based on published data, and their RNA expression levels compared in TCGA data from 304 CC and 3 normal adjacent tissue samples. There were 18 differentially expressed TCA cycle-related genes identified, with a threshold of *p* < 0.05 and |log_2_FC > 1.5|, of which 17 (PKM, GPI, IDH1, SHMT1, MTHFD1, SHMT2, ENO1, IDH2, ALDOA, DHFR, ELOVL3, SCD, TYMS, HK2, ALDOB, and PKLR) were upregulated and only one gene, ACAT1, was downregulated in tumor tissues (Figure 1A). Correlations among the mRNA expression levels of TCA cycle-related DEGs were analyzed by Pearson correlation analysis (Figure 1B). The results showed that all the TCA cycle-related DEGs had a positive correction with each other. In particular, FASN was significantly correlated with SCD (r = 0.66, *p* < 0.001) and PKM was significantly correlated with ENO1 (r = 0.63, *p* < 0.001). Construction of a PPI network revealed that the top 5 genes including PKM, IDH1, ENO1, PKLR and IDH2 were selected, based on their values of closeness, to be the hub nodes in the PPI network (Figure 1C).

### Consensus clustering based on TCA cycle-related DEGs

**FIGURE 2:**
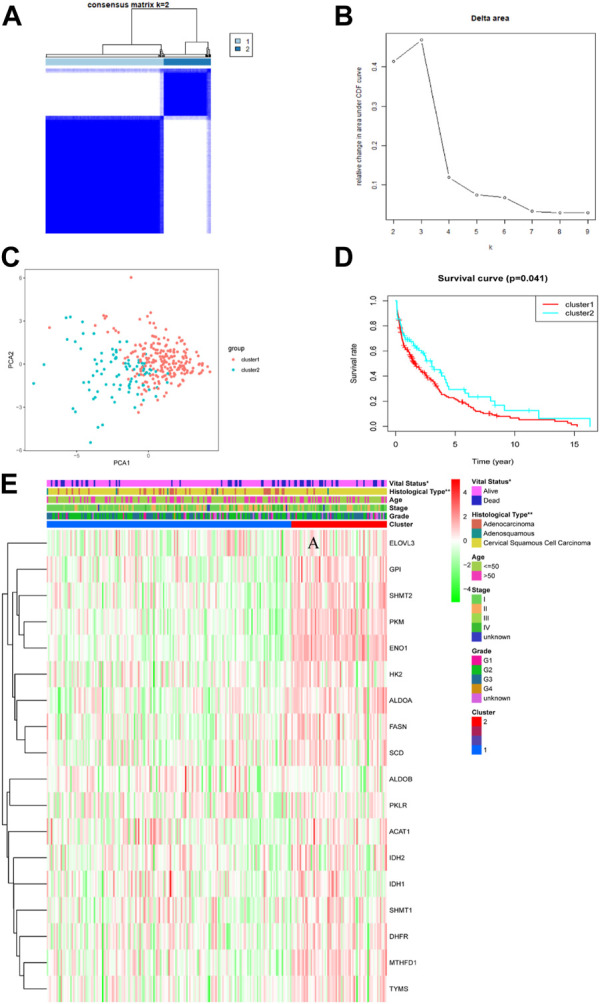
Tumor subclusters based on the TCA cycle-related DEGs. **(A)** Consensus clustering matrix for *k* = 2. **(B)** Delta area value for k = 2, 3, 4, 5, 6, 7, 8, and 9. **(C)** PCA analysis between different subclusters (red: cluster 1; green: cluster 2). **(D)** Kaplan–Meier curves of OS in two clusters. **(E)** Distribution heat map of seven prognostic TCA cycle-related genes and clinicopathological variables in the cluster1 and cluster 2 (*p* < 0.01 = **, and *p* < 0.05 = *).

To explore the relationships between CC subtypes and expression of the 18 TCA cycle-Related DEGs, consensus clustering analysis was performed to classify tumors according to expression levels of TCA cycle -related DEGs. Clustering variable (k) values from 2 to 9 were applied; when k = 2, intragroup correlations were low. Hence, patients with CC could be divided into two different subtypes, including 215 cases in cluster 1 and 87 cases in cluster 2 (Figures 2A, B). PCA was conducted to verify the ability of the model to group patients in the entire set and observed that patients in different clusters were dispersed in two directions (Figure 2C). There was a significant difference in OS time between the two clusters (*p* = 0.041) (Figure 2D). Further, the associations between the clustering and clinicopathological parameters were examined. The significant difference was found between cluster 1 and cluster 2, for the survival state (*p* < 0.05) and pathological type (*p* < 0.01). In contrast, other parameters such as age, tumor grade and clinical stage were no significant different (Figure 2E).

### Construction of Prognostic Signature for TCGA CC

**FIGURE 3:**
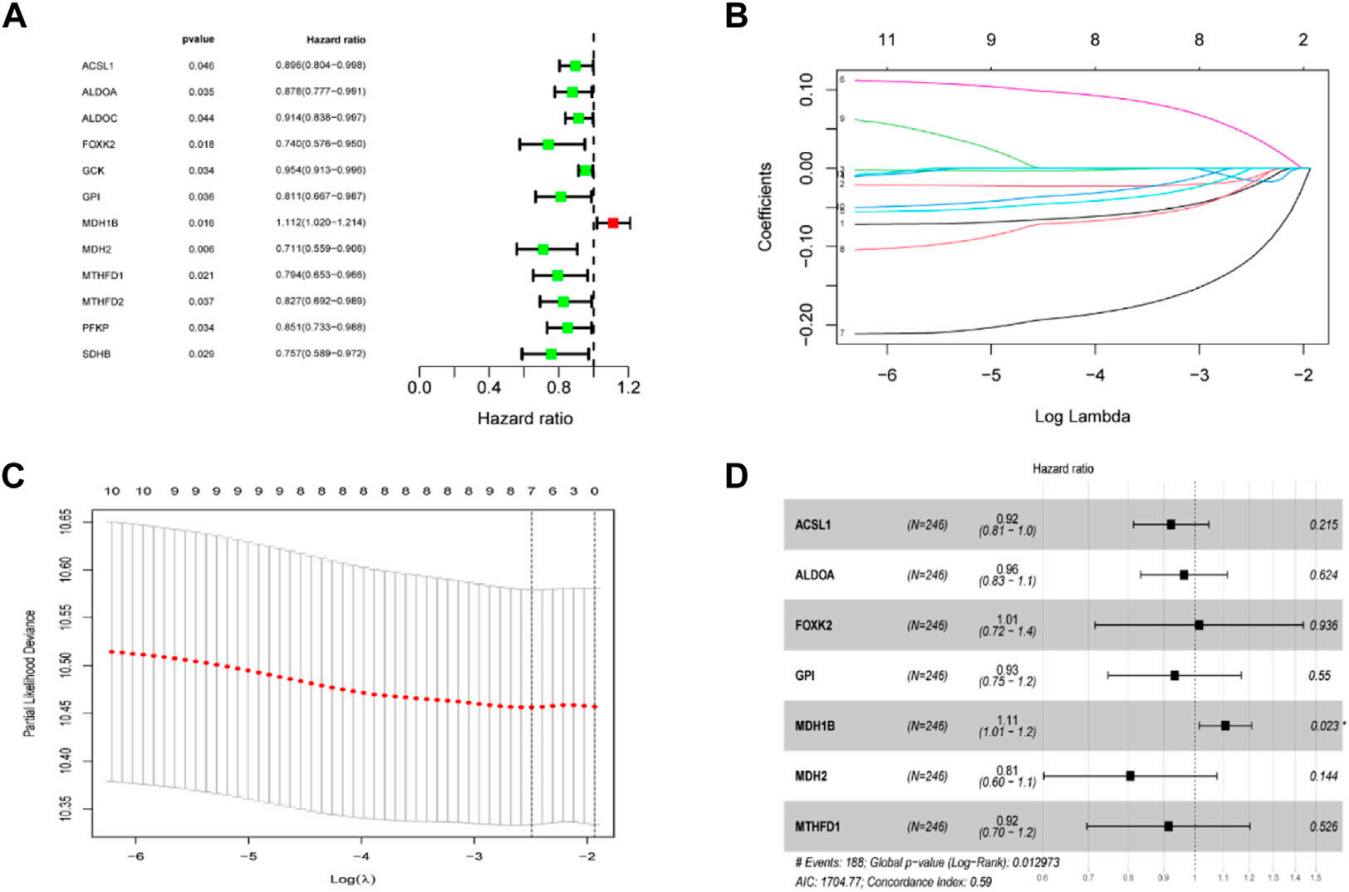
Establishment of the TCA cycle-related signature in the TCGA dataset. **(A)** Univariate cox regression analysis screened prognostic TCA cycle-related genes (*p* < 0.05). **(B)** LASSO regression of the 12 prognostic genes. **(C)** Cross-validation for tuning the parameter selection in the LASSO regression. **(D)** Stepwise multivariate cox regression analysis showed 7 independent prognostic genes.

The TCA cycle-related genes were all chosen for the univariate Cox regression analysis, and we found that 12 genes were significantly associated with OS in TCGA CC (Figure 3A). A LASSO regression analysis was applied to establish a prognostic gene signature using the 12 genes mentioned above. Following LASSO analysis to minimize overfitting (Figures 3B,C), seven genes involving ACSL1, ALDOA, FOXK2, GPI, MDH1B, MDH2 and MTHFD1 were identified (Figure 3D).

**FIGURE 4:**
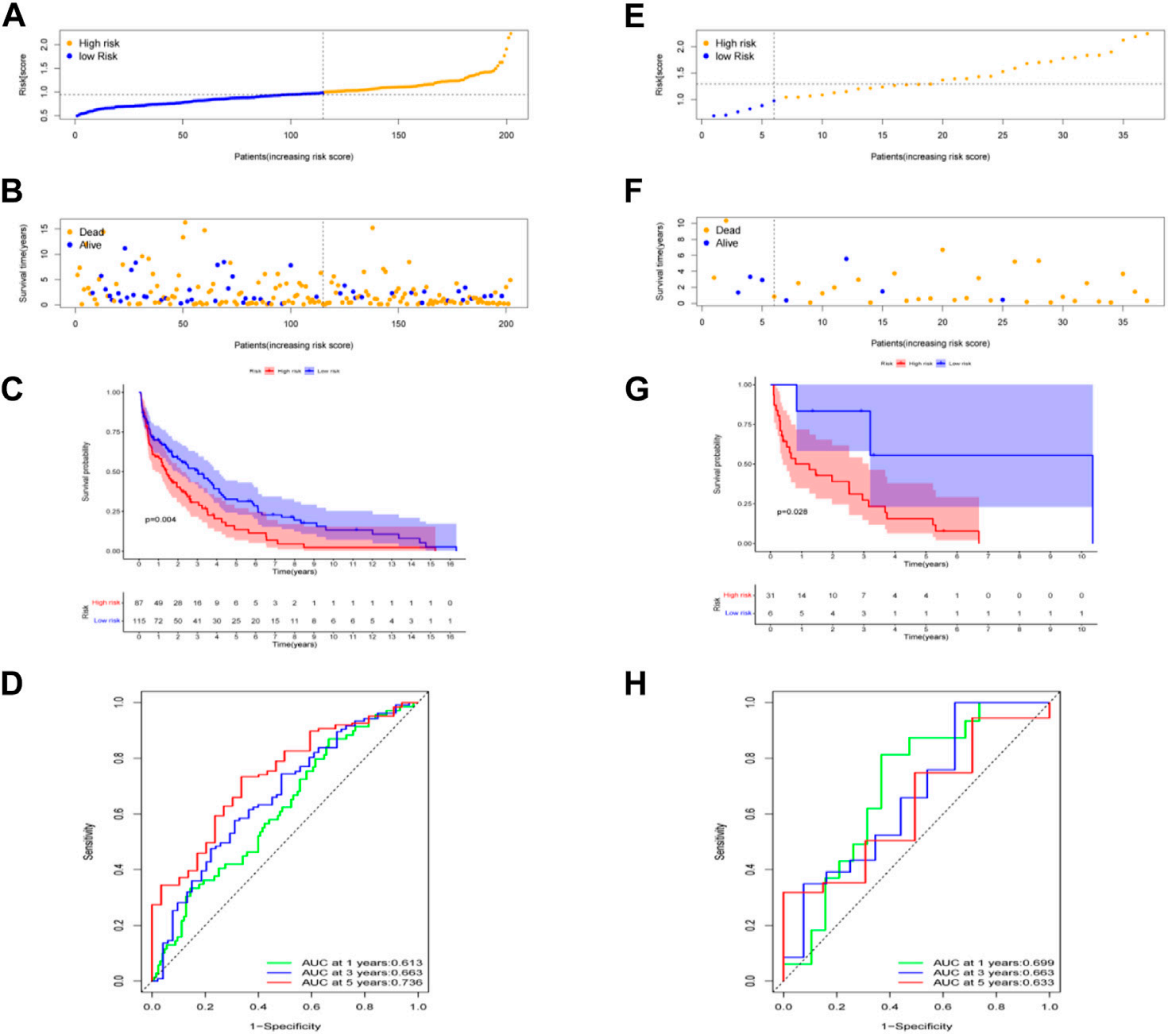
Prognostic value of the risk patterns of the signature in the TCGA dataset and prognosis analysis in different pathological types of cervical cancer: cervical squamous cell carcinoma in **(A–D)** and cervical adenocarcinoma in **(E,F)**. **(A,E)** Distribution of risk score. **(B,F)** Survival status plot and survival time. **(C,G)** Kaplan-Meier analysis of OS. **(D,H)** ROC curves analysis between high- and low-risk groups.

The risk score of seven genes was also calculated for further univariate and multivariate Cox regression analyses. The risk score formula to predict OS was developed as follows: risk score = (−0.080 × ACSL1) + (−0.036 × ALDOA)+ (0.014 × FOXK2) + (−0.068 × GPI) + (0.102 × MDH1B) + (−0.216 × MDH2) + (−0.089 × MTHFD1). It is well-known that survival times vary among patients with different pathological types of CC. Thus, prognosis analysis of the seven genes in different pathological types of CC including cervical squamous cell carcinoma and cervical adenocarcinoma. Using this signature, patients were further classified into equal high- and low-risk groups, based on the median risk value (Figures 4A, E). As illustrated in the scatter diagram in Figures 4B, F, individuals in the high-risk score group had worse outcomes than those in the low-risk group. In addition, a significant difference in OS time was detected between the two groups by Kaplan-Meier analysis (*p* < 0.01, *p* < 0.05) (Figures 4C, G). ROC curve analysis was conducted to evaluate the sensitivity and specificity of the prognostic model, resulting in AUC values of the models for predicting 1-, 3-, and 5-year OS of 0.613, 0.663, and 0.736 in cervical squamous cell carcinoma, while 1-, 3-, and 5-year OS of 0.699, 0.663, and 0.633 in cervical adenocarcinoma respectively (Figures 4D,H).

### External validation of the seven-gene signature

**FIGURE 5:**
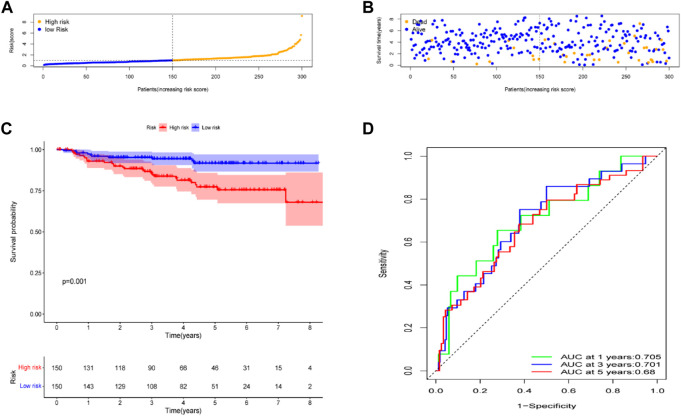
Validation of the risk model in the GEO cohort. **(A)** Distribution of risk score. **(B)** Survival status plot and survival time. **(C)** Kaplan-Meier analysis of OS. **(D)** ROC curves analysis between high- and low-risk groups.

To test the robustness of the gene signature model built from the TCGA data, data from 300 patients with CC in the GEO cohort, the GSE44001 dataset were also divided into high- and low-risk groups using a similar formula to TCGA data (Figure 5A). According to the uniform formula, the survival analyses found that patients with higher risk scores had poorer OS (*p* = 0.001) (Figures 5B, C). In the GSE44001 dataset, the AUC was 0.705 at one year, 0.701 at three years and 0.68 at five years (Figure 5D).

### Functional enrichment analysis

**FIGURE 6:**
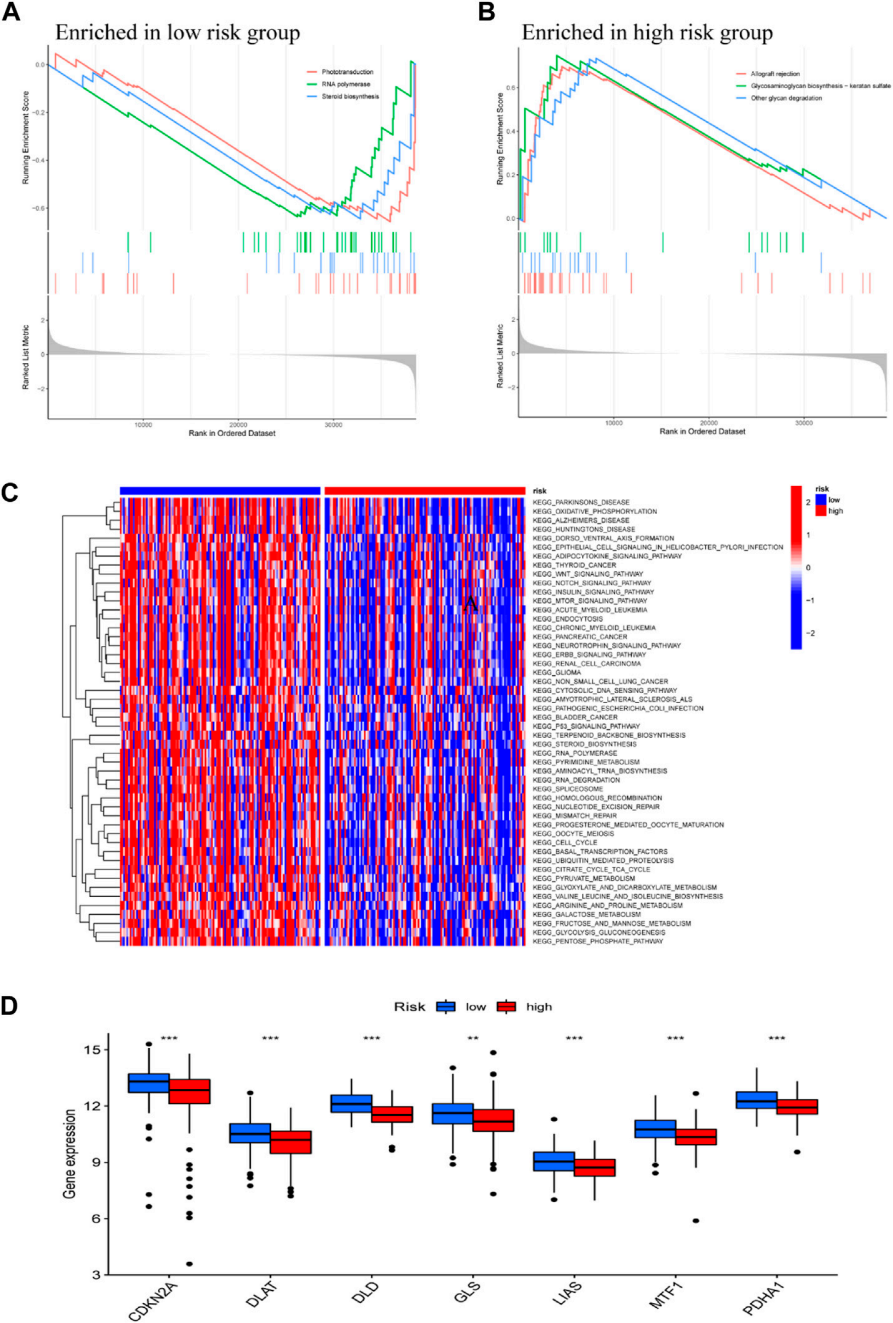
Functional Analysis DEGs between low- and high-risk subgroups. GSEA KEGG pathway enrichment in low-risk group **(A)**, and high-risk group **(B)**. **(C)** The heatmap of KEGG pathways between low- and high-risk subgroups analyzed by GSVA. **(D)** Box plot of cuproptosis-related genes analysis based on low- and high-risk subgroups; *p < 0.05, **p < 0.01, ***p < 0.001.

To explore the potential biological processes in high- and low-risk groups, we performed a GSEA. The KEGG pathway analysis showed that phototransduction, RNA polymerase, and steroid biosynthesis were mainly enriched in the low-risk group (Figure 6A), while allograft rejection, glycosaminoglycan biosynthesis—keratan sulfate and other glycan degradation were principally enriched in the high-risk group (Figure 6B). To further identify the expression difference of these two groups, the GSVA enrichment analysis revealed that cancer pathways, including Wnt, Notch and mTOR signaling pathway were highly expressed in low-risk group, compared with high-risk group (Figure 6C). These results suggest that Metabolic reprogramming modulates tumor proliferation, apoptosis, and cell cycle *via* these pathways. The box plot of Cuproptosis-related gene analysis illustrated that in the low-risk group, CDKN2A DLAT DLD GLS LIAS MTF1, and PDHA1 were significantly downregulated in the high-risk group (Figure 6D). The finding echoed the definition of the novel cell deathmodality “Cuproptosis” which is featured by disturbing specific mitochondrial metabolic enzymes (Tsvetkov et al., 2022).

### Correlation between the predictive signature and CC therapy

**FIGURE 7:**
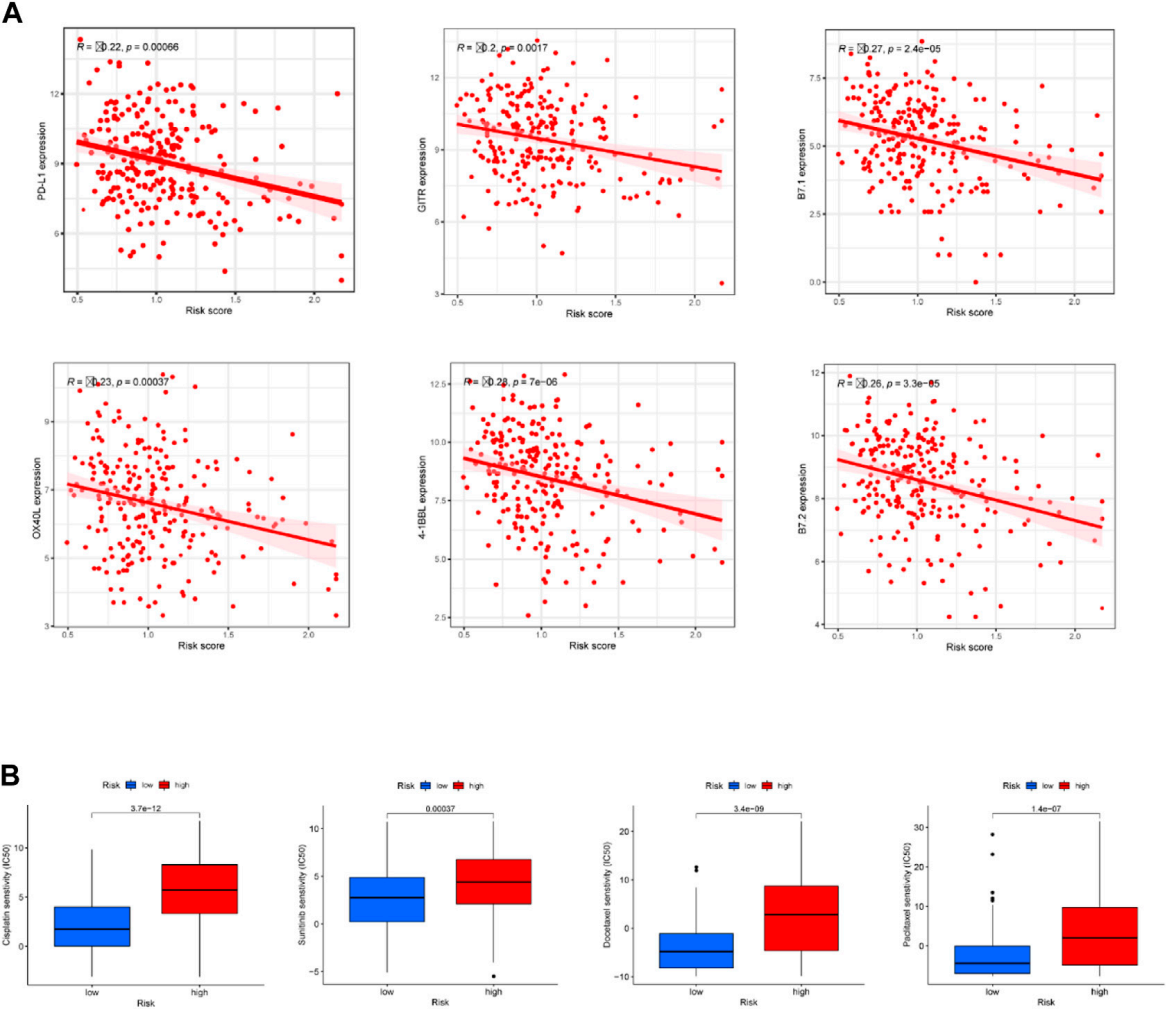
Comparison of treatment drugs sensitivity between high- and low-risk groups. **(A)** Correlation analysis of the expressions of six immune checkpoints with riskscore. **(B)** IC50 of cisplatin, sunitinib, docetaxel and paclitaxel in high and low risk groups.

Correlation assessment of the association between risk score and immune checkpoint-related genes found that PD-L1, 4-1BBL, OX40L, GITR, B7.1, and B7.2 had a negative correction with the risk score (Figure 7A). In other words, CC patients with higher risk scores had lower expression levels of these immune checkpoints. Our data suggest that patients in low-risk group may be more sensitive to immunotherapy. In addition to immunotherapy, we also analyzed the association between the predictive signature and the efficacy of general chemotherapy for CC. The results found that the IC50 of sunitinib, paclitaxel, cisplatin, and docetaxel was lower in the low-risk group (Figure 7B), which is helpful for exploring individualized treatment schemes suitable for high- and low-risk group patients.

### Real-time quantitative reverse transcription PCR (qRT-PCR).

**FIGURE 8:**
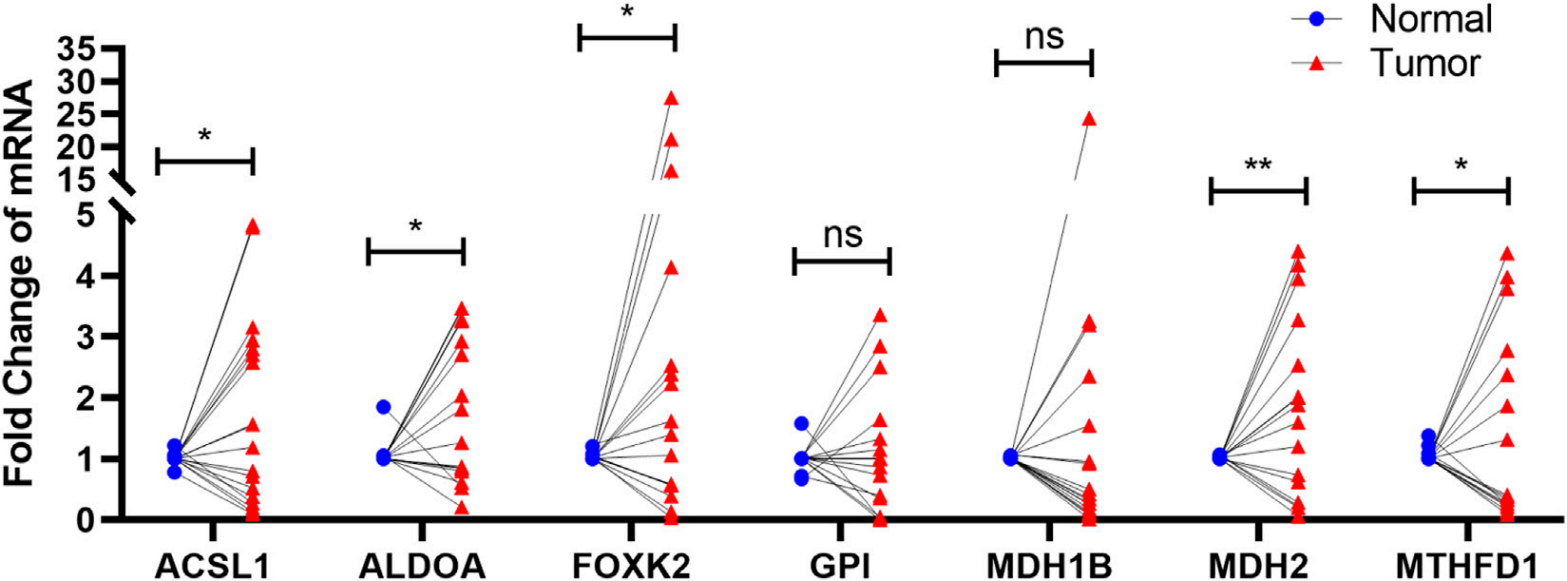
qRT-PCR The results showed that the expression of ACSL1, ALDOA, FOXK2, MDH2, and MTHFD1 in cervical cancer was significantly expressed compared with normal group. ns p > 0.05; ∗p < 0.05; ∗∗p < 0.01.

To determine whether the seven prognostic genes were differentially expressed in CC tissues, a total of 19 paired clinical CC tissues and adjacent normal tissues were analyzed each gene expression using qRT-PCR. The findings illustrated that the expression levels of ACSL1, ALDOA, FOXK2, MDH2, and MTHFD1 in cervical cancer specimens were differentially expressed in contrast with those in normal specimens, whereas there was no significant difference of the expression levels of GPI and MDH1B (Figure 8).

## Discussion

CC with high incidence and mortality rate remains a considerable health burden in females worldwide. The occurrence and development of CC is a complex, multi-step and multi-gene process, among which high-risk human papillomavirus persistent infection is the main factor (Crosbie et al., 2013). Previous studies stress the importance of TCA cycle in cancer because its products influence cell viability and proliferation (DeBerardinis and Chandel, 2016; Kim and DeBerardinis, 2019). Further, accumulating evidence to illustrate that metabolic heterogeneity influences therapeutic vulnerabilities and may predict clinical outcomes (Eniafe and Jiang, 2021). It used to be thought that cancer progression bypass TCA cycle which is in accord with Warburg effect. However, such concept has been challenged and may be revised with the increasing studies demonstrated that TCA cycle is of great importance in cancers. TCA cycle also generates energy and building blocks to meet the need of cancer cells growth, but hyperactivation of TCA cycle was previously considered to produce excess reaction oxygen species that is otherwise toxic to cells.

One study showed that through AMPK-mediated PDHA phosphorylation, the TCA cycle drives cancer cells to adapt to the metastatic microenvironment for metastasis (Cai et al., 2020). Besides, recent reports also demonstrated that certain TCA intermediates, such as oxaloacetate (OAA) and ketoglutarate (a-KG), play an important role in ROS detoxification (Sawa et al., 2017). Altogether, TCA cycle play a non-negligible role in tumorigenesis, metastasis and therapy. Therefore, we constructed an innovative signature based on TCA cycle associated genes. The results suggested that the TCA cycle related signature have substantial value for predicting OS and the drug sensitivity in CC.

The signature was comprised of seven core genes involving ACSL1, ALDOA, FOXK2, GPI, MDH1B, MDH2 and MTHFD1. FOXK2 was targeted by TP53TG1 *via* regulating miR-33a-5p and with the involvement of PI3K/AKT/mTOR signaling pathway to accelerates the CC development (Liao et al., 2022). The mechanism study confirmed that circ-ITCH regulated the expression of FOXK2 by adsorbing microrRNA-93-5p (miR-93-5p) to inhibit tumor growth (Li et al., 2020). Notably, FOXK2 was upregulated in the high-risk group in our model. Except for FOXK2, other genes involved in the model have not previously been studied in the context of CC. ACSL1, encoding an isozyme of the long-chain fatty-acid-coenzyme in a ligase family, is downregulate by MiR-27a-3p and MiR-205 to increase the risk of liver cancer and hepatocellular carcinoma respectively (Cui et al., 2014; Sun et al., 2020; Quan et al., 2021). Similarly, ACSL1 acted as a protective factor in our prognostic model. ALDOA increased most markedly in response to TGF-β and further the results of *in vitro* and *in vivo* experiments show that ALDOA is associated with the proliferation and metastasis of pancreatic cancer cells (Ji et al., 2016). GPI, a member of the glucose phosphate isomerase protein family, can be used as a potential biomarker for predicting OS of hepatocellular carcinoma (Lyu et al., 2016). Numerous studies have shown that the overexpression of GPI/AMF is connected with poor prognosis, such as tumor invasion and the increased mortality in many cancer types, including gastrointestinal (Gong et al., 2005), kidney, lung and breast cancers (Baumann et al., 1990; Nabi et al., 1991; Jiang et al., 2006). MDH1B, (Malate Dehydrogenase 1B) is one of alleles encoding MDH isozymes. Carm1-mediated arginine methylation of MDH1 inhibits glutamine metabolism, thereby inhibiting the growth of pancreatic cancer (Wang et al., 2016). The tumor-suppressive effects of methyl 3-(3-(4-(2,4,4-trimethylpentan-2-yl)phenoxy)propanamido)benzoate were investigated and demonstrated that dual inhibition of MDH1 and MDH2 is an effective approach to target tumor metabolism (Naik et al., 2017). As a metabolism-related enzyme, MDH2 is overexpressed in endometrial carcinoma tissues and correlated with its grade. These results demonstrated that MDH2 promoted cancer progression of endometrial cancer (Zhuang et al., 2017). Studies have found that MTHFD1 deficiency can significantly inhibit the antioxidant defense ability of cells and inhibit the distant metastasis of tumors, which indicates that the high expression of MTHFD1 in liver cancer tissues indicates a poor prognosis.

In this study, we calculated a risk score based on the constructed prognostic model, and classified patients with cervical cancer into high-risk and low-risk groups according to the median of this risk score. The Kaplan-Meier survival curve showed that the OS of the high-risk group was significantly lower than that of the low-risk group. The calculation of the AUC value showed the value of the risk signature in predicting survival prognosis. Validation set based on the GEO database was analyzed with similar methods to verify the stability of the predictive model. It is important to note that chemotherapy is the main treatment approach for CC, but it often causes a number of side effects, and cancer cells can become resistant to chemotherapy (Grasso et al., 2017; Choi et al., 2021). Cancer treatment failure in CC can be attributed to drug resistance. Thus, assessment of individual drug response is crucial in the treatment of CC. Accumulating evidence suggest that this chemoresistance is strongly associated with specific metabolic abnormalities in cancer cells, particularly increased use of glucose and the amino acid glutamine that promotes anabolic processes. (Luo et al., 2009; Vivanco, 2014; Belizario et al., 2016). In fact, Reprogramming of metabolic pathways in cancer cells is a complicated and confusing process. A popular view holds that a key function of oncogenes is to reprogram cellular metabolism back to the building blocks that maintain unrestrained tumor growth (Yao et al., 2008). In our study, we developed an integrated computational approach to identify metabolic reprogramming of multiple drugs based on TCA cycle related genes.

Finally, we carried out qRT-PCR on the seven TCA cycle associated genes linked to the prognoses of CC patients. These results demonstrated the accuracy of our first step of difference analysis, improved the credibility of subsequent studies, and also confirmed the association of risk genes with TCA cycle, further validating the predictive power of our model.

Although this study found that TCA cycle related pathways affect the progression and treatment of CC, there remain some limitations. Firstly, the small number of normal samples in TCGA database may lead to a certain bias in the analysis. Secondly, in order to explore the direct mechanisms additional *in vitro* and *in vivo* studies are necessary. Finally, this study is designed as a retrospective study, and a large number of experimental data are needed to confirm the study results.

## Conclusion

In conclusion, our study revealed that the prognostic model based on TCA cycle associated genes are significantly correlated with the survival and clinicopathological characteristics in CC. TCA cycle related signature are effective biomarkers for predicting the prognosis of CC patients.

## Data Availability

The original contributions presented in the study are included in the article/Supplementary Material, further inquiries can be directed to the corresponding authors.

## Abbreviations

CC, cervical cancer; TCA cycle, tricarboxylic acid cycle; DEGs, differentially expressed genes; GSVA, gene set variation analysis; GSEA, gene set enrichment analysis; TCGA, The Cancer Genome Atlas; GEO, Gene Expression Omnibus; OS, overall survival; AUC, area under the curve; ROC, receiver operating characteristic; GO, gene ontology; KEGG, Kyoto Encyclopedia of Genes and Genomes; IC50, half-maximal inhibitory concentration; MSigDB, Molecular Signatures Database; PCA, Principal Component Analysis.
